# Sexual and drug use risk behaviour trajectories among people treated for recent HCV infection: the REACT study

**DOI:** 10.1002/jia2.26168

**Published:** 2023-09-07

**Authors:** Joanne M. Carson, Sebastiano Barbieri, Evan Cunningham, Eric Mao, Marc van der Valk, Jürgen K. Rockstroh, Margaret Hellard, Arthur Kim, Sanjay Bhagani, Jordan J. Feld, Ed Gane, Maria C. Thurnheer, Julie Bruneau, Elise Tu, Gregory J. Dore, Gail V. Matthews, Marianne Martinello, the REACT study group

**Affiliations:** ^1^ Kirby Institute UNSW Sydney Sydney New South Wales Australia; ^2^ The Centre for Big Data Research in Health UNSW Sydney Sydney New South Wales Australia; ^3^ Division of Infectious Diseases Amsterdam Infection and Immunity Institute University Medical Centers University of Amsterdam Amsterdam The Netherlands; ^4^ Stichting HIV Monitoring Amsterdam The Netherlands; ^5^ University Hospital Bonn Bonn Germany; ^6^ Burnet Institute Melbourne Victoria Australia; ^7^ The Alfred Hospital Melbourne Victoria Australia; ^8^ Massachusetts General Hospital Boston Massachusetts USA; ^9^ Royal Free Hospital London UK; ^10^ Toronto Centre for Liver Diseases Toronto General Hospital Toronto Ontario Canada; ^11^ Auckland City Hospital Auckland New Zealand; ^12^ Department of Infectious Diseases Bern Inselspital Bern Switzerland; ^13^ Research Center Centre Hospitalier de l'Université de Montréal Montréal Quebec Canada

**Keywords:** HCV, HIV, STI, GBM, PWID, reinfection

## Abstract

**Introduction:**

Exploration of sexual and drug use behaviours following treatment for recent hepatitis C virus (HCV) is limited. This analysis modelled behavioural trajectories following treatment for recent HCV and assessed reinfection.

**Methods:**

Participants treated for recent HCV in an international trial (enrolled 2017–2019) were followed at 3‐monthly intervals for up to 2 years to assess longitudinal behaviours. Population‐averaged changes were assessed using generalized estimating equations. Distinct behavioural trajectories were identified using group‐based trajectory modelling. HCV reinfection incidence was calculated using person‐years (PY) of observation.

**Results:**

During the follow‐up of 212 participants (84% gay and bisexual men [GBM]; 69% HIV; 26% current injecting drug use [IDU]), behavioural trajectories for IDU and stimulant use (past month) did not change. However, population‐averaged decreases in the likelihood of daily IDU (adjusted odds ratio [AOR] 0.83; 95% CI 0.72, 0.95) and opioid use (AOR 0.84; 95% CI 0.75, 0.93) were observed. Among GBM, behavioural trajectories for chemsex did not change. Population‐averaged decreases in condomless anal intercourse with casual male partners (CAI‐CMP) (AOR 0.95; 95% CI 0.90, 0.99) and group‐sex (AOR 0.86; 95% CI 0.80, 0.93) were observed, but masked distinct trajectories. While a proportion had a decreased probability of CAI‐CMP (23%) and group‐sex (59%) post‐treatment, a substantial proportion retained a high probability of these behaviours. High HCV reinfection incidence was observed for the sustained high probability IDU (33.0/100 PY; 95% CI 17.7, 61.3) and chemsex (23.3/100 PY; 95% CI 14.5, 37.5) trajectories.

**Conclusions:**

Limited sexual and drug use behavioural change was observed following treatment for recent HCV, supporting access to surveillance and (re)treatment.

## 1 INTRODUCTION

The development of highly effective direct‐acting antiviral therapies has transformed the clinical management of hepatitis C virus (HCV) infection and led to reductions in global prevalence [1, 2, 3]. However, most countries are not on track to meet World Health Organization HCV elimination targets, with an estimated 1.5 million people newly infected every year [3].

In high‐income countries, HCV transmission occurs primarily among people who inject drugs [4] and gay and bisexual men (GBM) with HIV [5, 6]. Modelling, supported by emerging real‐world data, has shown treating those at the highest risk of HCV transmission will be essential to achieve sustained declines in HCV incidence [7, 8, 9, 10, 11]. Treating people with recent HCV represents an opportunity to interrupt transmission in the context of ongoing sexual or drug use risk behaviours. However, HCV cure and the benefits of treatment may be transitory if risk behaviours continue post‐treatment and reinfection occurs.

The aim of this analysis was to assess longitudinal sexual and drug use behaviours following treatment for recent HCV infection and the impact of risk behaviour trajectories on HCV reinfection and sexually transmitted infection (STI) incidence.

## 2 METHODS

### 2.1 Study design

The Recently Acquired HCV Infection Trial (REACT) was an international randomized non‐inferiority trial evaluating the efficacy of sofosbuvir‐velpatasvir for 6 or 12 weeks among people with recent HCV (primary infection or reinfection; duration of infection <12 months). REACT recruited participants through 24 sites internationally (Australia [*n* = 5], Canada [*n* = 4], Germany [*n* = 4], Netherlands [*n* = 1], New Zealand [*n* = 1], Switzerland [*n* = 3], United Kingdom [*n* = 4] and United States [*n* = 2]) between February 2017 and July 2019. Methodology REACT has been described previously [12]. All participants entered post‐treatment follow‐up with visits scheduled every 3 months for up to 2 years (until March 2020) to evaluate treatment response, monitor for reinfection and assess longitudinal risk behaviours.

To be included in this analysis, participants must have completed a behavioural questionnaire at one or more pre‐treatment study visits and one or more post‐treatment study visits.

### 2.2 Procedures

Participants completed a self‐administered behavioural questionnaire at screening, baseline and all post‐treatment visits (including end‐of‐treatment [ETR], sustained virological response post‐treatment week 12 [SVR12] and for up to 21 months thereafter). Questionnaires collected information on demographics, drug and alcohol use, opioid agonist treatment (OAT), other drug health services accessed (psychosocial, rehabilitation and withdrawal management) and self‐reported testing/diagnosis of STIs. Among GBM, questionnaires collected additional information on sexual behaviours. HCV RNA tests were performed at each study visit.

Participants provided written informed consent. The study protocol was approved by the St Vincent's Hospital, Sydney Human Research Ethics Committee, and local ethics committees and was conducted according to the Declaration of Helsinki and International Conference on Harmonisation Good Clinical Practice guidelines. The study was registered with clinicaltrials.gov (NCT02625909).

### 2.3 Definitions

Reinfection was defined as quantifiable HCV RNA after ETR with an HCV strain distinct from the primary infecting strain (confirmed by sequencing) or quantifiable HCV RNA after SVR12. Group‐sex was defined as sex involving three or more individuals. Chemsex (sexualized drug use) was defined as condomless anal intercourse and concurrent injecting or non‐injecting drug use (IDU) involving methamphetamine, mephedrone, γ‐hydroxybutyrate, γ‐butyrolactone and ketamine. Serosorting was defined as selectively engaging in condomless anal intercourse with other men of the same HIV and/or HCV status. Alcohol use was assessed using the Alcohol Use Disorders Identification Test–Consumption (AUDIT‐C; score range, 1–12) [2]. Scores of three or more (women) and four or more (men) indicated hazardous consumption. Social functioning was assessed using the Social Functioning Questionnaire [3] (SFQ; score range, 0–24). Higher scores indicate poorer social functioning. Sharing of injecting equipment included sharing of needles, syringes or ancillary injecting equipment.

### 2.4 Measures

Three behavioural outcomes were evaluated in relation to the treatment of recent HCV in the overall population: IDU, stimulant use (cocaine, methamphetamine, mephedrone and synthetic cathinones) and opioid use (heroin, illicit synthetic opioids and prescription opioids). Three behavioural outcomes were evaluated among GBM: condomless anal intercourse with casual male partners (CAI‐CMP), group‐sex and chemsex.

### 2.5 Statistical analyses

Population‐averaged changes in behaviours over time were estimated using a generalized estimating equation (GEE) extension of logistic regression with a binomial family function and logit link for binary variables. Models estimated the effect of time since enrolment on each outcome using odds ratio (OR) with 95% confidence interval (CI). Time effect was assessed in incremental study visits, irrespective of time lapses between visits. Drug use models were adjusted for OAT, sex, age, sexual identity, HIV and country of residence. Sexual behaviour models among GBM were adjusted for age, country and HIV.

As population‐averaged behavioural assessments can mask distinct trajectories of behaviour within a population, group‐based trajectory modelling (GBTM) was used to identify homogeneous clusters of behaviour that may remain stable or change over time [13, 14]. For each behavioural outcome, the number of groups (trajectories) and their shape were informed by previous studies examining sexual and drug use behaviours [15, 16, 17, 18] and several statistical criteria. For each outcome, the final number of groups was determined by selecting the model that minimized the Akaike Information Criterion, minimized the Bayesian Information Criterion, maximized average posterior probability of group membership and where group membership was more than 5% of the population [13, 14, 19, 20]. Parsimonious models were obtained by excluding polynomial terms (trajectory shapes) that did not attain statistical significance (*p*<0.05). Trajectories were described as high (sustained behaviour [average probability >0.5 at each visit]), moderate (stable but not regular behaviour [average probability ≤0.5 and >0.1 at each visit]), fluctuating (non‐linear behavioural changes over time [variational range exceeding >0.3 over the course of the study]) or low (no or rare occurrence of this behaviour [average probability <0.1 at each visit]).

HCV reinfection and STI incidence were calculated for the behavioural probability trajectories assigned during the GBTM procedure using person‐time of observation and were reported as number of cases per 100 person‐years (PY). CI for rates were calculated using Poisson distribution. Multiple HCV reinfection and STI events during the follow‐up period were included. HCV reinfection and STI incidence were compared between trajectory groups using incidence rate ratios. Kaplan−Meier cumulative hazards for HCV reinfection and STI incidence were plotted for trajectory groups to illustrate the distribution of incident events over the study period and groups compared using log‐rank testing.

### 2.6 Sensitivity analysis

To minimize selection bias due to loss to follow‐up, missing visits or questionnaire non‐completion, we developed a conservative definition of study retention *a priori* (seven visits) and refit the GEE models. Missing values due to non‐response were infrequent (<5% for any variable) and were left as is.

### 2.7 Software

Statistical analyses were performed in R version 2.4.0 (using geepack, poissonirr, survival packages). GBTM was performed in Stata version 14.2 (using the traj package).

## 3 RESULTS

### 3.1 Characteristics of study participants

Of 222 participants enrolled, 212 (95%) completed at least one pre‐ and one post‐treatment behavioural questionnaire and were included in this analysis (Table 1). Most identified as gay or bisexual men (84%), had HIV (69%) and were treated for recent primary HCV infection (64%). Almost half (47%) had used drugs (mainly stimulants [38%]) and 26% had injected drugs in the month prior to enrolment. Among people who injected drugs in the past month (*n* = 56), most GBM reported injecting less than weekly (60%), whereas most heterosexual men and women reported injecting more than weekly (54%). When comparing the characteristics of heterosexual men (*n* = 25) and women (*n* = 8), the only significant difference was a younger median age for women 31 versus 47 years (*p* = 0.03). Among GBM (*n* = 179), most identified as gay (96%), reported CAI‐CMP (80%) and group‐sex (64%) in the past month and almost one‐third reported chemsex (31%).

**Table 1:** Enrolment characteristics of REACT participants treated for recent HCV infection

	Gay and bisexual men (*n* = 179)	Women and heterosexual men (*n* = 33)	Total (*n* = 212)
Age (years, median [IQR])	43 [37, 51]	44 [35, 54]	43 [36, 52]
Male sex, *n* (%)	179 (100.0)	25 (75.8)	204 (96.2)
Race, *n* (%)			
Black	1 (0.6)	4 (12.1)	5 (2.4)
White	151 (84.4)	26 (78.8)	177 (83.5)
Asian	8 (4.5)	1 (3.0)	9 (4.2)
Mixed race	9 (5.0)	0 (0.0)	9 (4.2)
Other or unknown	10 (5.6)	2 (6.1)	12 (5.7)
Hispanic ethnicity, *n* (%)	13 (7.3)	1 (3.0)	14 (6.6)
Sexual orientation, *n* (%)			
Bisexual	6 (3.4)	1 (3.0)	7 (3.3)
Gay/lesbian	173 (96.6)	0 (0.0)	173 (81.6)
Heterosexual/straight	0 (0.0)	31 (93.9)	31 (14.6)
Not stated	0 (0.0)	1 (3.0)	1 (0.5)
Country of residence, *n* (%)			
Australia	17 (9.5)	5 (15.2)	22 (10.4)
Canada	7 (3.9)	9 (27.3)	16 (7.5)
Germany	56 (31.3)	0 (0.0)	56 (26.4)
Netherlands	28 (15.6)	0 (0.0)	28 (13.2)
New Zealand	0 (0.0)	2 (6.1)	2 (0.9)
Switzerland	8 (4.5)	5 (15.2)	13 (6.1)
United Kingdom	56 (31.3)	2 (6.1)	58 (27.4)
United States	7 (3.9)	10 (30.3)	17 (8.0)
Higher education, *n* (%)	101 (56.4)	3 (9.1)	104 (49.1)
Full or part‐time employment, *n* (%)	134 (74.9)	5 (15.2)	139 (65.6)
Psychiatric co‐morbidity, *n* (%)			
None	154 (86.0)	20 (60.6)	174 (82.1)
Anxiety	3 (1.7)	3 (9.1)	6 (2.8)
Depression	22 (12.3)	4 (12.1)	26 (12.3)
Bipolar disorder, schizophrenia or psychosis	0 (0.0)	6 (18.2)	6 (2.8)
History of incarceration, *n* (%)	7 (3.9)	22 (66.7)	29 (13.7)
Social functioning score (median [IQR])	7 [5, 11]	16 [12, 24]	8 [5, 13]
Recent HCV infection, *n* (%)			
Primary infection	117 (65.4)	18 (44.5)	135 (63.7)
Reinfection	62 (34.6)	15 (45.5)	77 (36.3)
Mode of HCV acquisition, *n* (%)			
IDU	16 (8.9)	26 (78.8)	42 (19.8)
Sexual—same sex exposure	154 (86.0)	0 (0.0)	154 (72.6)
Sexual—opposite sex exposure	6 (3.4)	4 (12.1)	10 (4.7)
Other	3 (1.7)	3 (9.1)	6 (2.8)
HIV infection, *n* (%)	140 (78.2)	7 (21.2)	147 (69.3)
Screened for STI in the past 12 months, *n* (%)	164 (91.6)	16 (48.5)	180 (84.9)
Diagnosed with STI in the past 12 months, *n* (%)	108 (60.3)	2 (6.1)	110 (51.9)
Ever injected drugs, *n* (%)	81 (45.3)	28 (84.8)	109 (51.4)
Injected drugs in the past 6 months, *n* (%)	53 (29.6)	22 (66.7)	75 (35.4)
Injected drugs in the past month, *n* (%)	43 (24.0)	13 (39.4)	56 (26.4)
Any illicit drug use in the past month, *n* (%)	75 (41.9)	25 (75.8)	100 (47.2)
Stimulant use in the past month, *n* (%)	64 (35.8)	16 (48.5)	80 (37.7)
Opioid use in the past month, *n* (%)	20 (11.2)	20 (60.6)	40 (18.9)
Other drug use in the past month, *n* (%)	17 (9.5)	6 (18.2)	23 (10.8)
Ever received opioid agonist treatment, *n* (%)	8 (4.5)	12 (36.4)	20 (9.4)
Currently receiving opioid agonist treatment, *n* (%)	0 (0.0)	1 (3.0)	1 (0.5)
Ever accessed other drug health services, *n* (%)	27 (15.1)	24 (72.7)	51 (24.1)
Alcohol use (AUDIT‐C score [median, IQR])	3 [1, 4]	2 [1, 4]	2 [1, 4]
CAI‐CMP in the past month, *n* (%)	144 (80.4)	NA	NA
Group‐sex in the past month, *n* (%)	114 (63.7)	NA	NA
Chemsex in the past month, *n* (%)	55 (30.7)	NA	NA

Abbreviations: CAI‐CMP, condomless anal intercourse with casual male partner; GBM, gay and bisexual men; HCV, hepatitis C virus; STI, sexually transmitted infection.

### 3.2 Population‐averaged changes and trajectories of drug use behaviours during and following HCV treatment

**Figure 1:**
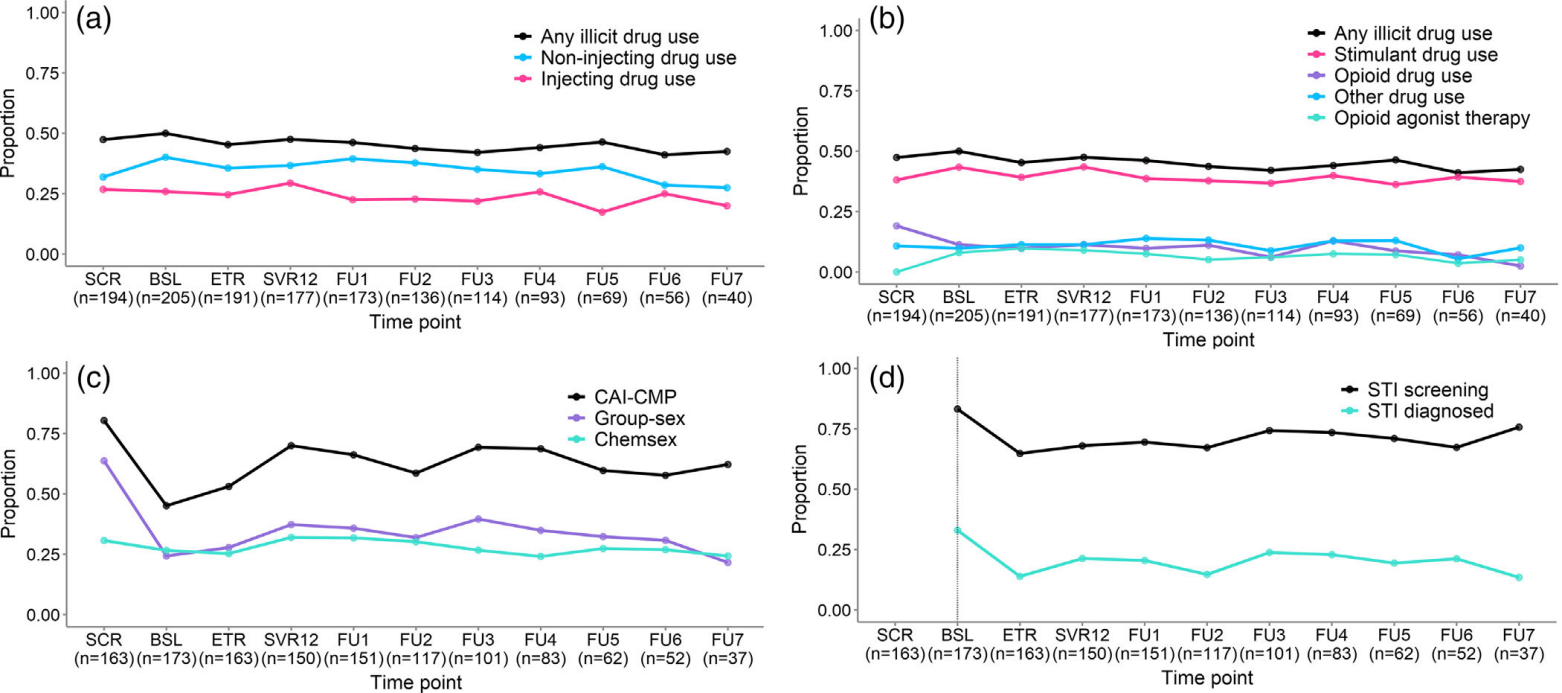
Proportion of participants reporting drug use and sexual risk behaviours. Proportion of participants reporting (A) injecting drug use and (B) type of drug used and opioid agonist treatment before, during and following treatment for recent HCV infection. Proportion of gay and bisexual men reporting (C) sexual risk behaviour and (D) STI diagnosis before, during and following treatment of recent HCV infection. Abbreviations: BSL, baseline; CAI‐CMP, condomless anal intercourse with casual male partner; ETR, end or treatment; FU, follow‐up; SCR, screening; STI, sexually transmitted infection; SVR12, sustained virological response 12‐weeks post treatment.

**Figure 2:**
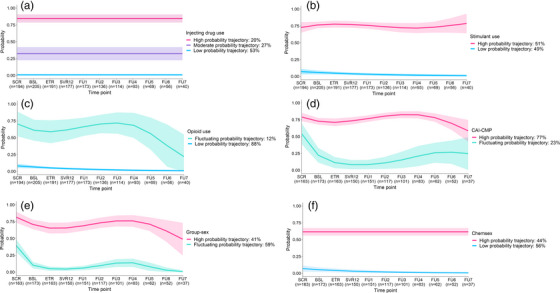
Group‐based trajectory modelling behavioural outcomes. Behavioural trajectories for (A) injecting drug use, (B) stimulant use and (C) opioid use before, during and following treatment for recent HCV infection in the overall population; behavioural trajectories for (D) condomless anal intercourse with casual male partners, (E) group‐sex and (F) chemsex before, during and following treatment for recent HCV infection among gay and bisexual men. Abbreviations: BSL, baseline; CAI‐CMP, condomless anal intercourse with casual male partner; ETR, end or treatment; FU, follow‐up; SCR, screening; SVR12, sustained virological response 12‐weeks post treatment.

During follow‐up, participants had a median of eight visits (IQR 6, 10) and contributed a total of 1448 observations. Figure 1 presents the proportion of participants reporting each drug use behavioural outcome. Table 2 presents the adjusted GEE analyses. Figure 2 presents the GBTM analyses for IDU, stimulant use and opioid use. Table S1 presents the drug use behaviour model selection process.

**Table 2:** Population‐averaged changes in drug use and sexual behaviours before, during and following treatment for recent HCV infection

Drug use behaviours (*n* = 212)	AOR^a^	*p*‐Value
Injection drug use	0.97 (0.93, 1.03)	0.452
Weekly IDU	0.93 (0.84, 1.02)	0.117
Daily IDU	0.83 (0.72, 0.95)	0.001
Sharing injecting equipment	1.01 (0.96, 1.07)	0.687
All stimulant use	1.00 (0.96, 1.05)	0.898
Stimulant IDU	1.00 (0.95, 1.06)	0.979
Stimulant non‐IDU	0.98 (0.94, 1.03)	0.938
All opioid use	0.84 (0.75, 0.93)	<0.001
Opioid IDU	0.87 (0.78, 0.98)	0.017
Opioid non‐IDU	0.96 (0.87, 1.06)	0.432
Opioid agonist treatment	1.19 (1.08, 1.30)	<0.001
Other drug use	1.01 (0.94, 1.08)	0.856
Polydrug use	0.97 (0.90, 1.03)	0.304

Sexual behaviours (*n* = 179)	AOR^b^	*p*‐Value
CAI‐CMP	0.95 (0.90, 0.99)	0.024
Group‐sex	0.84 (0.80, 0.89)	<0.001
Chemsex	0.97 (0.93, 1.02)	0.251
STI screening^c^	0.97 (0.91, 1.03)	0.389
STI diagnosis^c^, ^d^	0.93 (0.87, 0.99)	0.004
Serosorting behaviour	0.92 (0.85, 1.01)	0.084

*Note*: Each row represents an adjusted model. The estimated odds ratio indicates the average behaviour change across two consecutive visits, irrespective of time lapses between visits.

Abbreviations: AOR, adjusted odds ratio; CAI‐CMP, condomless anal intercourse with casual male partner; IDU, injecting drug use; STI, sexually transmitted infection.

^a^ Adjusted for sex, sexual identity, HIV, country and OAT.

^b^ Adjusted for age, country and HIV.

^c^ Calculated from baseline visit due to discrepancies in reporting periods for screening and post‐screening visits.

^d^ STI diagnosis also adjusted for STI screening.

IDU did appear to change significantly during follow‐up in population‐averaged analysis. In behavioural modelling, three distinct probability trajectories were identified: high (20%), moderate (27%) and low (53%). IDU behavioural trajectories remained stable over time. Those assigned to the high and moderate IDU probability trajectories (vs. low) had poorer social functioning (12 vs. 9 vs. 8; *p* = 0.008), education (23% vs. 43% vs. 59%; *p* = 0.002) and employment (17% vs. 62% vs. 76%; *p*<0.001). Most GBM were assigned to the low (58%) and moderate (26%) IDU probability trajectories. While IDU in the last month did not appear to change, population‐averaged decreases in daily injecting were observed, with each incremental visit associated, on average, with a 17% decrease in odds of daily injecting.

A population‐averaged decrease in opioid use was also observed, with each incremental study visit associated, on average, with a 16% decrease in odds of all opioid use (statistically significant for injecting, but not for non‐injecting, opioid use). In behavioural modelling, two distinct probability trajectories were identified: fluctuating (12%) and low (88%). A decrease in the likelihood of opioid use was observed in the fluctuating probability trajectory; however, this was non‐statistically significant (*p* = 0.088). Those assigned to the fluctuating (vs. low) opioid use probability trajectory had higher stimulant use (58% vs. 35%; *p* = 0.047) and poorer social functioning (13 vs. 8; *p*<0.001), lower education (13% vs. 54%; *p*<0.001) and employment (29% vs. 70%; *p*<0.001). Notably, 97% of GBM were assigned to the low opioid use probability trajectory. A population‐averaged increase in OAT was observed, with each incremental study visit associated, on average, a 19% increase in the odds of receiving OAT. However, the number of people receiving OAT was small (*n* = 19).

Stimulant use did not appear to change significantly during follow‐up in population‐averaged analysis. In behavioural modelling, two distinct probability trajectories were identified: high (51%) and low (49%). Stimulant use trajectories remained relatively stable over time. A lower proportion of those assigned to the high (vs. low) stimulant use probability trajectory were employed (57% vs. 74%; *p* = 0.012).

For those assigned to the high IDU probability trajectory, 98% were also assigned to the high stimulant use probability trajectory and 27% to the fluctuating opioid use probability trajectory. Among those assigned to high‐probability trajectories for IDU and stimulant use, the average probability of these behaviours at each visit were 0.85 and 0.75, respectively.

### 3.3 Population‐averaged changes and trajectories of sexual behaviours among GBM following HCV treatment

During follow‐up, GBM had a median of seven visits (IQR 5, 9) and contributed a total of 1252 observations. Figure 1 presents the proportion of participants reporting each sexual behavioural outcome. Table 2 presents the adjusted GEE analyses. Figure 2 presents the GBTM analyses for CAI‐CMP, group‐sex and chemsex. Table S2 presents the sexual behaviour model selection process.

Modest population‐averaged decreases in CAI‐CMP were observed, with each incremental study visit associated on average with a 5% decrease in CAI‐CMP. In behavioural modelling, two distinct probability trajectories were identified: high (77%) and fluctuating (23%). The fluctuating probability trajectory showed a rapid decrease in the probability of CAI‐CMP following enrolment with an increase following post‐treatment week 12 (coincident with SVR assessment [cure]). A higher proportion of those assigned to the high (vs. fluctuating) CAI‐CMP probability trajectory reported group‐sex (73% vs. 53%; *p* = 0.012) at enrolment.

A pronounced population‐averaged decrease in group‐sex was observed, with each incremental study visit associated, on average, with a 16% decrease in odds of group‐sex. In behavioural modelling, two distinct probability trajectories were identified: high (41%) and fluctuating (59%). The fluctuating probability trajectory showed a decreasing likelihood of group‐sex following enrolment, with a gradual increase post‐treatment. There also appeared to be a slight decline in the likelihood of group‐sex for those assigned to the high‐probability trajectory following enrolment and during treatment. A higher proportion of those assigned to the high (vs. fluctuating) group‐sex probability trajectory had an STI diagnosed in the last 12 months (73% vs. 53%; *p* = 0.012) at screening.

Chemsex did not appear to change significantly during follow‐up in population‐averaged analysis. In behavioural modelling, two distinct probability trajectories were identified: high (44%) and low (56%). Chemsex behavioural trajectories remained stable over time. Those assigned to the high (vs. low) chemsex probability trajectory were more likely to have injected drugs in the month prior to enrolment (44% vs. 26%; *p* = 0.021), have accessed other drug health services (23% vs. 10%; *p* = 0.024) and been treated for recent HCV reinfection (48% vs. 5%; *p*<0.001).

For those assigned to the high chemsex probability trajectory, 92% were also assigned to the high CAI‐CMP probability trajectory, 56% to the high group‐sex probability trajectory and 36% to the high IDU probability trajectory. Among those assigned to the high probability trajectories for CAI‐CMP, group‐sex and chemsex, the average probability of these behaviours at each visit were 0.75, 0.69 and 0.62, respectively.

### 3.4 Incidence and risk of HCV reinfection

**Figure 3:**
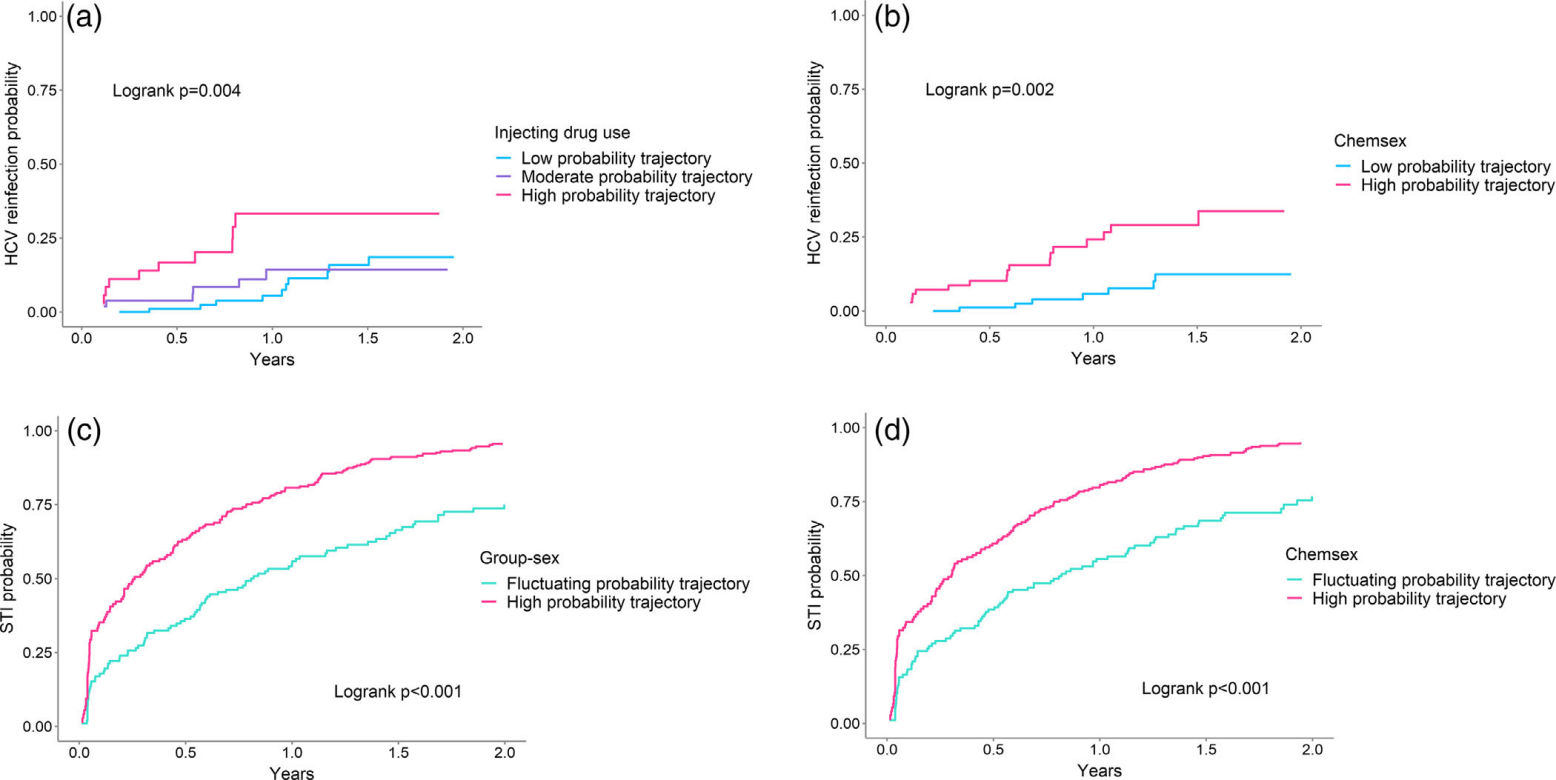
Cumulative hazards curves for behavioural trajectories. Cumulative hazard of HCV reinfection for (A) injecting drug use and (B) chemsex behavioural trajectories, and, of sexually transmitted infections for (C) group‐sex and (D) chemsex behavioural trajectories.

Overall, 26 cases of reinfection were identified among 25 participants (265 PY; median 11 months [IQR 6, 17]). HCV reinfection incidence was 13.2/100 PY (95% CI 9.0, 19.3), increasing significantly to 33.0/100 PY (95% CI 17.7, 61.3) for those assigned to the high IDU probability trajectory (Table 3). Cumulative hazard of HCV reinfection was significantly higher among individuals assigned to high IDU probability trajectory compared to those assigned to the moderate or low probability trajectories (*p* = 0.004; Figure 3). No cases of HCV reinfection were reported among individuals assigned to the fluctuating opioid use probability trajectory.

**Table 3:** HCV reinfection and STI incidence rates and risk for the assigned sexual and drug use risk trajectory groups

Probability trajectory	HCV reinfection incidence rate/100 PY^a^	HCV reinfection incidence rate ratio	*p*‐Value	STI incidence rate/100 PY^b^	STI incidence rate ratio	*p*‐Value
OVERALL						
(*n* = 212)	13.2 (9.0, 19.3)			103.2 (90.7, 117.5)		
IDU						
Low	9.0 (4.8, 16.8)	–	–	95.8 (79.9, 114.8)	–	–
Moderate	10.6 (4.8, 23.6)	1.23 (0.45, 3.38)	0.689	113.9 (89.9, 144.2)	1.31 (0.97, 1.76)	0.075
High	33.0 (17.7, 61.3)	4.32 (1.79, 10.38)	0.001	110.1 (81.6, 148.4)	1.14 (0.80, 1.62)	0.460
Stimulant use						
Low	9.1 (4.7, 17.5)	–		74.4 (59.6, 92.7)	–	–
High	17.2 (10.7, 27.7)	1.98 (0.88, 4.44)	0.097	129.8 (110.6, 152.3)	1.66 (1.26, 2.17)	<0.001
Opioid use						
Low	14.9 (10.1, 21.8)			108.8 (95.3, 124.2)	–	–
Fluctuating	0.0			48.6 (31.2, 124.6)	0.58 (0.31, 1.09)	0.093

GAY AND BISEXUAL MEN						
(*n* = 179)	13.8 (9.3, 20.6)			111.8 (98.1, 127.4)		
CAI‐CMP						
Fluctuating	4.8 (1.2, 19.2)	–	–	67.1 (45.3, 99.3)	–	–
High	16.6 (10.9, 25.2)	3.64 (0.86, 15.49)	0.080	121.9 (106.1, 140.0)	1.50 (0.99, 2.28)	0.055
Group‐sex						
Fluctuating	11.6 (6.6, 20.3)	–	–	74.8 (60.5, 92.3)	–	–
High	17.2 (9.8, 30.2)	1.55 (0.69, 3.44)	0.287	161.1 (136.4, 190.2)	1.94 (1.48, 2.54)	<0.001
Chemsex						
Low	6.9 (3.3, 14.6)	–	–	79.1 (64.0, 97.7)	–	–
High	23.3 (14.5, 37.5)	4.08 (1.69, 9.83)	0.002	150.2 (127.2, 177.3)	1.90 (1.46, 2.49)	<0.001

Abbreviations: CAI‐CMP, condomless anal intercourse with casual male partner; IDU, injecting drug use; PY, person‐years; STI, sexually transmitted infection.

^a^ HCV reinfection incidence calculated from end of DAA treatment: 194/212 had at least 2 visits post‐treatment with behavioural data and HCV RNA testing available.

^b^ STI incidence calculated from screening visit: 192/212 with at least 2 visits post‐screening with behavioural data and STI screening available.

Among GBM, 24 cases of reinfection were identified among 23 participants (173 PY; median 11 months [IQR 6, 17]). HCV reinfection incidence was 13.8/100 PY (95% CI 9.0, 19.3), increasing significantly to 23.3/100 PY (95% CI 14.5, 37.5) among the high chemsex probability trajectory (Figure 3). Cumulative hazard of HCV reinfection was significantly higher among individuals assigned to the high‐probability chemsex trajectory compared to those assigned to the low‐probability trajectory (*p* = 0.002; Figure 3).

Among women and heterosexual men, two cases of reinfection were identified among 28 participants (24 PY; median 9 months [IQR 5, 14]). HCV reinfection incidence was 8.4/100 PY (95% CI 2.1, 33.7).

### 3.5 Incidence and risk of STI

Overall, 232 cases of STI were reported among 102 participants (222 PY; median 13 months [IQR 8, 21]) (Table 3). STI incidence was 103.2/100 PY (95% CI 90.7, 117.5), increasing significantly to 129.8/100 PY (95% CI 110.6, 152.3) for those assigned to the high stimulant use probability trajectory.

Among GBM, 225 cases of STI were reported among 99 participants (201 PY; median 14 months, [IQR 9, 22]). STI incidence was 111.8/100 PY (95% CI 99.8, 127.4), increasing to 161.1/100 PY (95% CI 136.4, 190.2) and 152.1/100 PY (95% CI 127.2, 177.3) for those assigned to the high group‐sex and chemsex probability trajectories, respectively.

Cumulative hazard of STI was significantly higher among individuals assigned to high probability group‐sex (*p*<0.001) or chemsex (*p*<0.001) trajectories compared to those assigned to low probability trajectories for these behaviours (Figure 3). Table S5 presents incidence and risk ratios for specific STIs.

### 3.6 Sensitivity analysis

Compared to participants classified as retained in follow‐up (*n* = 134), those not retained were younger (median 41 vs. 45 years; *p* = 0.045), more likely to report opioid use (27% vs. 14%; *p* = 0.035) and less likely to report CAI‐CMP (69% vs. 86%) or be diagnosed with an STI (37% vs. 60%; *p* = 0.002; Table S3). In GEE analyses restricted to participants retained in follow‐up, ORs remained largely unchanged (Table S4).

## 4 DISCUSSION

This analysis assessed longitudinal sexual and drug use behaviours following treatment for recent HCV in an international cohort of predominantly GBM with HIV. Limited behavioural change was seen during and following HCV treatment. High rates of HCV reinfection were observed among participants with a sustained high probability of IDU and chemsex behaviours. These findings support regular reinfection surveillance and rapid access to retreatment for GBM and people who inject drugs treated for recent HCV.

Treatment of recent HCV infection provides an opportunity to reduce HCV transmission. However, for most of this cohort, engagement in HCV treatment did not appear to modify behaviour. While population‐averaged decreases in CAI‐CMP and group‐sex were observed among GBM, these masked distinct trajectories of behaviour within the population. Modelling of these sexual behaviours revealed a decreased probability among a minority (23%; with gradual increases observed post‐treatment), while most (77%) retained a high probability throughout. These findings differ from a study reporting changes no change in population‐averaged sexual behaviour among GBM treated for chronic HCV, although this study did not identify distinct trajectories of behaviour within the population [11]. It is possible that transient changes in sexual behaviour observed between screening and baseline in this study reflect the recency of HCV diagnosis and concern over onward transmission. No changes in chemsex behaviours were observed during or following treatment. A high incidence of HCV reinfection (23/100 PY) was observed among those with a sustained high probability of chemsex behaviour. These findings not only demonstrate the individual and population level importance of treating people with recent HCV infection, but the necessity of ensuring post‐treatment reinfection surveillance and retreatment (if required).

Trajectories for most drug use behaviours did not change during and following treatment. A high incidence of HCV reinfection (33/100 PY) was observed for those with a sustained high probability of IDU. Consistent with previous analyses, population‐averaged decreases in the likelihood of daily injecting and opioid use were observed and were likely associated with increased uptake of OAT among a small group of women and heterosexual men [16, 21]. Of note, no cases of HCV reinfection were reported among those with a decreasing probability of opioid use. Stimulant use was predominant in this cohort, reflecting the drug use preferences of the enrolled GBM. Consistent with a recent analysis among people who inject drugs treated for chronic HCV, no significant changes in stimulant use were observed [16]. Limited change in stimulant use may be attributable to a lack of treatment options. However, GBM may also have different motivations for stimulant use, including chemsex [22]. GBM reporting sexualized drug use may not engage with traditional harm reduction and prevention messaging, given a historical focus on people who use opiates. There is a critical need for increased HCV awareness, education and targeted harm reduction strategies influenced by drug type, drug use frequency and the social context in which drug use occurs [23, 24, 25].

There are marked differences in reinfection risk among individuals treated for recent and chronic HCV. The overall HCV reinfection incidence reported in this analysis (13/100 PY) was twice as high as that reported in recent meta‐analyses among GBM with HIV (6/100 PY) and people who inject drugs (6/100 PY) [26, 27]. However, most studies included in these meta‐analyses were among people treated for chronic HCV, with limited exploration of reinfection risk in the context of recent HCV and sexualized drug use [26, 27]. The overall STI incidence observed in this study was high (112/100 PY), but consistent with other studies conducted among GBM reporting high‐risk sexual behaviours [28, 29]. Also consistent, was the heightened risk of STI among those engaging in group‐sex and chemsex behaviours [30, 31]. No association between STI incidence and HCV incidence was observed. However, a correlation between decreased probability of CAI‐CMP and group‐sex with STI incidence was apparent.

Strengths of this analysis include the prospective study design, inclusion of key affected populations, collection of detailed sexual and drug use behavioural data, and the use of GBTM to identify distinct behavioural trajectories within the population. This analysis type represents a novel methodology to identify groups with sustained high‐risk behaviours and assess HCV reinfection and STI risk. The prospective study design allowed for frequent (3‐monthly) behavioural assessment and HCV RNA testing, limiting recall bias and improving reinfection detection. Limitations of this analysis included follow‐up time and study population. Duration of follow‐up was limited to that stipulated in the original trial protocol, with a median follow‐up of 1 year; study closure prematurely terminated follow‐up for some participants. STI diagnosis was self‐reported and reporting of STI type was incomplete for 36%. Behavioural trajectories and HCV/STI incidence were measured simultaneously. While this provided descriptive results on incidence rates in relation to key behavioural patterns, it did not allow for the examination of temporality. For some risk behaviours (including injecting frequency and injecting equipment sharing), the proportion of the population engaging in these behaviours was too small to model; however, population‐averaged changes were reported. While drug behaviour data were collected for all participants, only those identifying as gay or bisexual men were asked to report specific sexual risk behaviours previously described as associated with HCV transmission in this sub‐population. Further, the relatively small sample size with participants spread across nine different countries and 24 distinct health services has limited more nuanced inter‐group comparisons at a country and site level, and between gender and sexual identities. The clinical trial was conducted in high‐income countries with the enrolment of predominantly white GBM with HIV who were already engaged in care, excluding some vulnerable individuals who might be at even greater risk of reinfection. Undetected and untreated (re)infections drive HCV epidemics. However, identifying individuals in the acute phase of HCV (re)infection is challenging, as onset is generally asymptomatic. This has resulted in significant selection bias towards those receiving regular HCV surveillance. Those who disengage with care following treatment for recent HCV may have high baseline risk behaviours and may be less likely to modify these behaviours. As such, the reinfection incidence reported in this study is likely an underestimation of the true reinfection incidence among people treated for recent HCV. Given the potential impact of undetected HCV acute (re)infections on HCV elimination efforts, future research should ensure a broader representation of vulnerable populations, including people who inject drugs, along with greater gender and racial diversity.

## 5 CONCLUSIONS

Treating people with recent HCV will be critical to reduce transmission and achieve elimination. With limited risk behaviour modification following treatment, regular reinfection surveillance and rapid access to retreatment will be critical to achieve sustained declines in the incidence of HCV among GBM and people who inject drugs.

## COMPETING INTERESTS

The authors declare no competing interests.

## AUTHORS’ CONTRIBUTIONS

MM and GVM proposed the study analysis. GVM and GJD designed the REACT study. GVM, GJD, MVdV, MH, AK, SaBh, JJF, EG, AR and JB were involved in participant recruitment and data collection. GVM, GJD, MVdV, MH, AK, SaBh, JJF, EG, AR, JB and MM provided study governance through the Protocol Steering Committee. ET oversaw study coordination.

JMC conducted the data analysis, with oversight from MM, SeBa and GVM. JMC drafted the manuscript with critical revision of the manuscript for important intellectual content by all authors. All authors have seen and approved the final version of the manuscript.

## FUNDING

The REACT study was funded by the National Institutes of Health. Study medication was provided by Gilead Sciences Inc. The Kirby Institute is funded by the Australian Government Department of Health and Ageing.

## DISCLAIMER

The views expressed in this publication do not necessarily represent the position of the Australian Government.

## Supporting information


**Table S1**: Model section criteria in group‐based trajectory modelling of drug use risk behaviours.
**Table S2**: Model section criteria in group‐based trajectory modelling of sexual risk behaviours among gay and bisexual men.
**Table S3**: Comparison of screening characteristics for participants classified as retained in follow up and those that were classified as not retained in follow‐up.
**Table S4**: Sensitivity analysis of population averaged changes in drug use and sexual risk behaviours before, during and following treatment for recent HCV infection.
**Table S5**: Specific STI incidence rates and hazard ratios for the assigned sexual and drug use risk trajectory groups. (A) Chlamydia and Gonorrhoea, (B) Syphilis and Unknown.

## Data Availability

Due to the sensitive nature of some of the data, including that related to IDU, data included in this manuscript have not been placed in an open‐access database. However, data are available to be shared on request to the protocol steering committee.
