# Supplementary material for: Sexual and drug use risk behaviour trajectories among people treated for recent HCV infection: the REACT study
Source: J Int AIDS Soc. 2023 Sep 7;26(9):e26168. doi: 10.1002/jia2.26168 (PMC10483502; doi:10.1002/jia2.26168)
Supplement: Supplementary file 1 — Table S1: Model section criteria in group‐based trajectory modelling of drug use risk behaviours. Table S2: Model section criteria in group‐based trajectory modelling of sexual risk behaviours among gay and bisexual men. Table S3: Comparison of screening characteristics for participants classified as retained in follow up and those that were classified as not retained in follow‐up. Table S4: Sensitivity analysis of population averaged changes in drug use and sexual risk behaviours before, during and following treatment for recent HCV infection. Table S5: Specific STI incidence rates and hazard ratios for the assigned sexual and drug use risk trajectory groups. (A) Chlamydia and Gonorrhoea, (B) Syphilis and Unknown. [file JIA2-26-e26168-s001.docx]

**Sexual and drug use risk behaviour trajectories among people treated for recent HCV infection: The REACT study**

Joanne M Carson^1^, Sebastiano Barbieri^2^, Evan Cunningham^1^, Eric Mao^1^, Marc van der Valk^3,4^, Jürgen K Rockstroh^5^, Margaret Hellard^6,7^, Arthur Kim^8^, Sanjay Bhagani^9^, Jordan J Feld^10^, Ed Gane^11^, Andri Rauch^12^, Julie Bruneau^13^, Elise Tu^1^, Gregory J Dore^1^, Gail V Matthews^1^, Marianne Martinello^1^ on behalf of the REACT study group

^1^Kirby Institute, UNSW Sydney, Sydney, Australia

^2^The Centre for Big Data Research in Health, UNSW Sydney, Sydney, Australia

^3^Division of Infectious Diseases, Amsterdam Infection and Immunity Institute, Amsterdam,

University Medical Centers, University of Amsterdam, The Netherlands

^4^Stichting HIV Monitoring, Amsterdam, the Netherlands

^5^University Clinic Bonn, Bonn, Germany

^6^Burnet Institute, Melbourne, Australia

^7^The Alfred Hospital, Melbourne, Australia

^8^Massachusetts General Hospital, Boston, USA

^9^Royal Free Hospital, London, United Kingdom

^10^Toronto Centre for Liver Diseases, Toronto General Hospital, Toronto, Canada

^11^Auckland City Hospital, Auckland, New Zealand

^12^Department of Infectious Diseases, Bern Inselspital, Bern, Switzerland

^13^Centre Hospitalier de l’Université de Montréal, Montréal, Canada

**Supplementary Table 1.** Model section criteria in group-based trajectory modelling of drug use risk behaviours

| **Variable** | **Model** | **No. of groups** | **Polynomial order^1^** | **BIC^2^ (n=212)** | **BIC^3^ (n=1448)** | **AIC^4^** | **APP^5^** |
| --- | --- | --- | --- | --- | --- | --- | --- |
| INJECTING DRUG USE |  |  |  |  |  |  |  |
|  | A | 1 | 3 | -820.29 | -824.13 | -813.58 | NA |
|  | B | 2 | 3 3 | -603.24 | -611.89 | -588.14 | 0.970 |
|  | C | 3 | 3 3 3 | -591.29 | -604.74 | -567.80 | 0.919 |
|  | D | 3 | 0 2 2 | -578.38 | -587.02 | -563.27 | 0.912 |
|  | **E** | **3** | **0 0 0** | **-568.40** | **-573.20** | **-560.00** | **0.906** |
| STIMULANT USE |  |  |  |  |  |  |  |
|  | A | 1 | 3 | -983.56 | -987.4 | -976.85 | NA |
|  | B | 2 | 3 3 | -685.95 | -694.6 | -670.84 | 0.980 |
|  | C | 3 | 3 3 3 | -677.76 | -691.21 | -654.26 | 0.886 |
|  | D | 3 | 0 1 3 | -667.28 | -675.92 | -652.17 | 0.895 |
|  | **E** | **2** | **0 3** | **-682.45** | **-689.18** | **-670.71** | **0.979** |
| OPIOID USE |  |  |  |  |  |  |  |
|  | A | 1 | 3 | -504.10 | -507.94 | -497.39 | NA |
|  | B | 2 | 3 3 | -412.92 | -421.57 | -397.82 | 0.970 |
|  | C | 3 | 3 3 3 | -409.19 | -422.64 | -385.70 | 0.897 |
|  | D | 2 | 1 3 3 | -405.15 | -416.67 | -385.01 | 0.925 |
|  | **E** | **2** | **1 3** | **-410.00** | **-416.73** | **-398.26** | **0.959** |

For each behaviour, the last row presents the final model.

^1^Polynomial order (trajectory shapes); 0 = zero-order; 1 = linear; 2 = quadratic, 3 = cubic

^2^BIC = Bayesian information criterion (for the total number of participants)

^3^BIC = Bayesian information criterion (for the total number of observations)

^4^AIC = Akaike information criterion

^5^APP = Average posterior probability of group membership

**Supplementary Table 2.** Model section criteria in group-based trajectory modelling of sexual risk behaviours among gay and bisexual men

| **Variable** | **Model** | **No. of groups** | **Polynomial order^1^** | **BIC^2^ (n=212)** | **BIC^3^ (n=1448)** | **AIC^4^** | **APP^5^** |
| --- | --- | --- | --- | --- | --- | --- | --- |
| CAI-CMP |  |  |  |  |  |  |  |
|  | A | 1 | 3 | -821.56 | -825.45 | -815.18 | NA |
|  | B | 2 | 3 3 | -750.56 | -759.31 | -736.22 | 0.971 |
|  | C | 3 | 3 3 3 | -750.61 | -764.23 | -728.30 | 0.842 |
|  | D | 3 | 0 3 3 | -738.01 | -748.71 | -720.48 | 0.885 |
|  | **E** | **2** | **3 3** | **-750.56** | **-759.31** | **-736.22** | **0.971** |
| GROUP-SEX |  |  |  |  |  |  |  |
|  | A | 1 | 3 | -805.32 | -809.21 | -798.95 | NA |
|  | B | 2 | 3 3 | -662.74 | -671.50 | -648.40 | 0.947 |
|  | C | 3 | 3 3 3 | -681.64 | -668.02 | -645.71 | 0.837 |
|  | D | 3 | 0 3 3 | -664.14 | -674.83 | -646.61 | 0.834 |
|  | **E** | **2** | **3 3** | **-662.74** | **-671.50** | **-648.40** | **0.947** |
| CHEMSEX |  |  |  |  |  |  |  |
|  | A | 1 | 3 | -760.92 | -764.81 | -754.55 | NA |
|  | B | 2 | 3 3 | -590.74 | -599.50 | -576.40 | 0.961 |
|  | C | 3 | 3 3 3 | -587.45 | -601.07 | -565.14 | 0.874 |
|  | D | 3 | 0 1 0 | -571.03 | -576.86 | -561.45 | 0.885 |
|  | **E** | **2** | **1 0** | **-579.38** | **-583.27** | **-573.00** | **0.961** |

For each behaviour, the last row presents the final model.

^1^Polynomial order (trajectory shapes); 0 = zero-order; 1 = linear; 2 = quadratic, 3 = cubic

^2^BIC = Bayesian information criterion (for the total number of participants)

^3^BIC = Bayesian information criterion (for the total number of observations)

^4^AIC = Akaike information criterion

^5^APP = Average posterior probability of group membership

**Abbreviations:** CAI-CMP, condomless anal intercourse with casual male partners

**Supplementary Table 3.** Comparison of screening characteristics for participants classified as retained in follow up and those that were classified as not retained in follow-up

| **Variable** | **Retained n=134** | **Not retained n=78** | **p-value** |
| --- | --- | --- | --- |
| Age, years [Median (IQR)] | **45 [39, 52]** | **41 [32, 52]** | **0.045** |
| Male sex, n (%) | 131 (97.8) | 73 (93.6) | 0.245 |
| Gay and bisexual men, n (%) | 118 (88.1) | 61 (78.2) | 0.087 |
| HIV, n (%) | 97 (72.4) | 50 (64.1) | 0.268 |
| **STI past 12 months, n (%)** | **81 (60.4)** | **29 (37.2)** | **0.002** |
| Injection drug use, past month, n (%) | 34 (25.4) | 22 (28.2) | 0.772 |
| Weekly IDU, past month, n (%) | 18 (8.5) | 7 (9.0) | 1.000 |
| Daily IDU, past month, n (%) | 7 (5.2) | 3 (3.8) | 0.904 |
| Injecting equipment sharing, n (%) | 25 (18.7) | 13 (16.7) | 0.858 |
| All stimulant use, past month, n (%) | 50 (37.3) | 30 (38.5) | 0.985 |
| Stimulant IDU, past month, n (%) | 27 (20.1) | 19 (24.4) | 0.586 |
| Stimulant non-IDU, past month, n (%) | 36 (26.9) | 23 (29.5) | 0.801 |
| **All opioid use, past month, n (%)** | **19 (14.2)** | **21 (26.9)** | **0.035** |
| Opioid IDU, past month, n (%) | 7 ( 5.2) | 7 ( 9.0) | 0.439 |
| Opioid non-IDU, past month, n (%) | 9 ( 6.7) | 5 ( 6.4) | 1.000 |
| Currently receives OAT, n (%) | 1 ( 0.7) | 0 ( 0.0) | 1.000 |
| Other drug use, past month, n (%) | 15 (11.2) | 8 (10.3) | 1.000 |
| Polydrug use, n (%) | 16 (11.9) | 18 (23.1) | 0.053 |
| Alcohol use, AUDIT-C score [Median, IQR] | 2.00 [1.00, 4.00] | 3.00 [1.00, 4.00] | 0.601 |
| **CAI-CMP past month, n (%)** | **102 (86.4)** | **42 (68.9)** | **0.009** |
| Group sex in past month, n (%) | 77 (57.5) | 37 (47.4) | 0.204 |
| Chemsex in past month, n (%) | 37 (27.6) | 21 (26.9) | 1.000 |
| Serosorting behaviour, n (%) | 12 (15.4) | 15 (11.2) | 0.503 |

**Supplementary Table 4.** Sensitivity analysis of population averaged changes in drug use and sexual risk behaviours before, during and following treatment for recent HCV infection

| **Drug use behaviours (n=134)** | **AOR*** | **p-value** |
| --- | --- | --- |
| Injection drug use | 0.98 (0.93, 1.03) | 0.380 |
| Weekly IDU | 0.91 (0.81, 1.01) | 0.081 |
| **Daily IDU** | **0.80 (0.68, 0.96)** | **0.014** |
| Sharing injecting equipment | 0.99 (0.93, 1.06) | 0.835 |
| All stimulant use | 1.00 (0.96, 1.04) | 0.963 |
| Stimulant IDU | 1.01 (0.95, 1.07) | 0.758 |
| Stimulant non-IDU | 0.99 (0.94, 1.04) | 0.662 |
| **All opioid use** | **0.88 (0.79, 0.99)** | **0.026** |
| Opioid IDU | 0.89 (0.79, 1.00) | 0.055 |
| Opioid non-IDU | 0.98 (0.88, 1.08) | 0.667 |
| **Opioid agonist treatment** | **1.19 (1.07, 1.33)** | **0.002** |
| Other drug use | 1.01 (0.94, 1.09) | 0.785 |
| Polydrug use | 1.01 (0.94, 1.08) | 0.841 |
| **Sexual behaviours (n=118)** | **AOR**** | **p-value** |
| CAI-CMP | 0.97 (0.92, 1.02) | 0.186 |
| **Group-sex** | **0.84 (0.79, 0.89)** | **<0.001** |
| Chemsex | 0.97 (0.92, 1.02) | 0.244 |
| STI screening^ | 0.97 (0.91, 1.04) | 0.376 |
| STI diagnosis^^#^ | 0.94 (0.87, 1.01) | 0.086 |
| Serosorting behaviour | 0.96 (0.87, 1.05) | 0.346 |

*Adjusted for sex, sexual identity, HIV, country, OAT

**Adjusted for age, country, HIV

^Calculated from baseline visit due to discrepancies in reporting periods for screening and post-screening visits

^#^STI diagnosis also adjusted for STI screening

Each row represents an adjusted model. The estimated odds ratio indicates the average behaviour change across 2 consecutive visits, irrespective of time lapses between visits.

**Abbreviations:** AOR: adjusted odds ratio; STI, sexually transmitted infection; CAI-CMP, condomless anal intercourse with casual male partners

**Supplementary table 5. Specific STI incidence rates and hazard ratios for the assigned sexual and drug use risk trajectory groups.** (A) Chlamydia and Gonorrhoea, (B) Syphilis and Unknown

**(A)**

| **Probability  trajectory** | **Chlamydia incidence  rate/100 PY^1^** | **Chlamydia  incidence rate ratio** | **p-value** | **Gonorrhoea incidence  rate/100 PY^1^** | **Gonorrhoea incidence  rate ratio** | **p-value** |
| --- | --- | --- | --- | --- | --- | --- |
| OVERALL (n=212) | 23.4 (17.9, 30.8) |  |  | 27.9 (21.8, 35.9) |  |  |
| IDU |  |  |  |  |  |  |
| Low | 25.4 (17.8, 36.1) | - | - | 20.5 (13.8, 30.3) | - | - |
| Moderate | 21.5 (12.5, 37.0) | 0.85 (0.44, 1.62) | 0.617 | 36.3 (23.9, 55.2) | 1.74 (0.98, 3.08) | 0.060 |
| High | 20.4 (10.2, 40.9) | 0.81 (0.37, 1.76) | 0.593 | 38.4 (23.1, 63.7) | 1.81 (0.95, 3.43) | 0.070 |
| Stimulant use |  |  |  |  |  |  |
| Low | 22.6 (15.1, 33.7) | - | - | 18.8 (12.1, 29.2) | - | - |
| High | 24.2 (16.7, 35.1) | 1.06 (0.61, 1.83) | 0.833 | 36.3 (26.9, 49.2) | 1.89 (1.11, 3.23) | 0.019 |
| Opioid use |  |  |  |  |  |  |
| Low | 24.4 (18.4, 32.2) | - | - | 29.8 (23.2, 38.4) | - | - |
| Decreasing | 14.6 (4.7, 45.2) | 0.59 (0.18, 1.89) | 0.372 | 9.7 (2.4, 38.8) | 0.32 (0.08, 1.30) | 0.110 |
| GBM (n=179) | 25.8 (19.7, 33.9) |  |  | 29.8 (23.1, 38.4) |  |  |
| CAI-CMP |  |  |  |  |  |  |
| Fluctuating | 16.1 (7.2, 35.8) | - | - | 10.7 (4.0, 28.6) | - | - |
| High | 28.0 (21.0, 37.4) | 1.77 (0.76, 4.15) | 0.188 | 34.1 (26.3, 44.4) | 3.28 (1.19, 9.06) | 0.022 |
| Group sex |  |  |  |  |  |  |
| Fluctuating | 20.0 (13.3, 30.0) | - |  | 25.2 (17.5, 36.3) | - | - |
| High | 33.6 (23.4, 48.4) | 1.69 (0.98, 2.92) | 0.061 | 35.9 (25.3, 51.1) | 1.44 (0.87, 2.39) | 0.160 |
| Chemsex |  |  |  |  |  |  |
| Low | 21.1 (14.0, 31.8) | - | - | 21.1 (14.1, 31.8) | - | - |
| High | 31.3 (21.8, 45.1) | 1.48 (0.86, 2.56) | 0.158 | 40.0 (29.0, 55.2) | 1.88 (1.11, 3.16) | 0.018 |

^1^STI incidence calculated from screening visit: 192/212 with at least 2 visits post-screening with behavioural data and STI screening available

**Abbreviations:** GBM, gay and bisexual men; CAI-CMP, condomless anal intercourse with casual male partners

**Supplementary table 5. Specific STI incidence rates and hazard ratios for the assigned sexual and drug use risk trajectory groups.** (A) Chlamydia and Gonorrhoea, (B) Syphilis and Unknown

**(B)**

| **Probability  trajectory** | **Syphilis  incidence  rate/100 PY^1^** | **Syphilis  incidence  rate ratio** | **p-value** | **Other/Unknown^2^ STI incidence  rate/100 PY^1^** | **Other/Unknown^2^ STI incidence rate ratio** | **p-value** |
| --- | --- | --- | --- | --- | --- | --- |
| OVERALL (n=212) | 16.2 (11.7, 22.5) |  |  | 37.0 (29.8, 45.9) |  |  |
| IDU |  |  |  |  |  |  |
| Low | 10.6 (6.2, 18.3) | - | - | 39.3 (29.6, 52.1) | - | - |
| Moderate | 26.4 (16.2, 43.1) | 2.74 (1.31, 5.69) | 0.007 | 34.5 (22.5, 53.0) | 0.96 (0.58, 1.61) | 0.891 |
| High | 17.9 (8.5, 37.6) | 1.67 (0.67, 4.19) | 0.272 | 33.5 (19.4, 57.9) | 0.84 (0.45, 1.55) | 0.155 |
| Stimulant use |  |  |  |  |  |  |
| Low | 8.5 (4.4, 16.2) | - | - | 24.5 (16.7, 35.9) | - | - |
| High | 23.4 (16.0, 34.1) | 2.60 (1.22, 5.53 | 0.013 | **48.4 (37.2, 62.9)** | **1.87 (1.17, 2.97)** | **0.008** |
| Opioid use |  |  |  |  |  |  |
| Low | 17.4 (12.5, 24.2) | - | - | 38.8 (31.0, 48.4) | - | - |
| Decreasing | 4.9 (0.7, 37.5) | 0.37 (0.05, 2.70) | 0.328 | 19.4 (7.3, 51.8) | 0.66 (0.24, 1.81) | 0.421 |
| GBM (n=179) | 17.4 (12.5, 24.2) |  |  | 40.2 (30.4, 50.0) |  |  |
| CAI-CMP |  |  |  |  |  |  |
| Fluctuating | 16.1 (7.2, 35.8) | - | - | 21.5 (10.7, 42.9) | - | - |
| High | 17.7 (12.3, 25.4) | 0.89 (0.80, 3.07) | 0.186 | 44.0 (35.4, 55.9) | 1.69 (0.81, 3.50) | 0.160 |
| Group-sex |  |  |  |  |  |  |
| Fluctuating | 13.0 (7.8, 21.6) | - | - | 18.3 (11.9, 28.0) | - | - |
| High | 23.2 (15.0, 35.9) | 1.50 (0.87, 2.60) | 0.186 | **69.5 (54.0, 89.6)** | **3.41 (2.07, 5.60)** | **<0.001** |
| Chemsex |  |  |  |  |  |  |
| Low | 10.1 (5.6, 18.2) | - |  | 25.4 (17.7, 36.6) | - | - |
| High | **25.9 (17.4, 38.6)** | **2.58 (1.27, 5.27)** | **0.009** | **59.5 (45.4, 78.1)** | **2.13 (1.35, 3.36)** | **0.001** |

^1^STI incidence calculated from screening visit: 192/212 with at least 2 visits post-screening with behavioural data and STI screening available

^2^Other STI includes Mycoplasma genitalium (2 x diagnosis), HPV (2 x diagnoses), Shigella (2 x diagnoses)

**Abbreviations:** GBM, gay and bisexual men; CAI-CMP, condomless anal intercourse with casual male partners; STI, sexual transmitted infection
